# Study on the Mechanism of Ionic Liquids Improving the Extraction Efficiency of Essential Oil Based on Experimental Optimization and Density Functional Theory: The Fennel (*Foeniculi fructus*) Essential Oil Case

**DOI:** 10.3390/molecules26113169

**Published:** 2021-05-26

**Authors:** Guolin Shi, Longfei Lin, Yuling Liu, Gongsen Chen, Anhui Yang, Yanqiu Wu, Yingying Zhou, Hui Li

**Affiliations:** 1 Institute of Chinese Materia Medica, China Academy of Chinese Medical Sciences, Beijing 100700, China; aidj1991@163.com (G.S.); linlongfei0417@126.com (L.L.); ylliu@icmm.ac.cn (Y.L.); gongsenchen@163.com (G.C.); yah1535@163.com (A.Y.); wyq1689926339@163.com (Y.W.); zhou1234y@163.com (Y.Z.); 2 School of Pharmacy, Jiangxi University of Traditional Chinese Medicine, Nanchang 330004, China

**Keywords:** essential oil, ionic liquids, multivariate analysis, multi-objective optimization, density functional theory

## Abstract

In this work, microwave-assisted ionic liquids treatment, followed by hydro-distillation (MILT-HD), as an efficient extraction technology, was used to extract essential oil. The purpose for this was to use multivariate analysis (MVA) models to investigate the effects of potential critical process parameters on the extraction efficiency of essential oil, and explore the mechanism of ionic liquids (ILs). According to the design of experiment (DoE), under optimal process conditions, the extraction efficiency of essential oil was dramatically enhanced, and had low energy demands. Since little is known regarding those mechanisms, according to the non-covalent interaction analysis, the underlying mechanism for ILs improving extraction efficiency was explored based on the density functional theory (DFT). The results showed that ILs could form intense non-covalent bond interaction with cellulose. It helped destroy the network hydrogen bond structure of cellulose in plant cells and caused the essential oils in the cells to be more easily exposed to the extraction solution, thereby accelerating extraction efficiency. Based on this work, it is conducive to understand the MILT-HD process better and gain knowledge of the mechanism of ILs.

## 1. Introduction

Due to the high added value of essential oils in the market, improving essential oil output will have great commercial values and benefits [1]. However, the traditional hydro-distillation (HD) method for extracting essential oil is inefficient, time-consuming, and energy-consuming. Another disadvantage is the low yield of essential oil and prolonged extraction duration, resulting in thermal degradations of liable components [2]. Consequently, it is necessary to adopt new technology to improve the extraction efficiency of essential oil from plants.

Compared with traditional molecular solvents, ionic liquids (ILs), composed of an organic cation and inorganic or organic anion, are green alternative solvents [3]. ILs have excellent properties, such as good thermal stability, low vapor pressure, non-flammability [4], and dissolving various biomass like cellulose [5]. Over the last several years, they have been considered as green additives to enhance essential oil yield [6]. Since ILs have excellent capacities for absorbing and transferring microwave energy effectively, ILs combined with microwave hydro-distillation can improve the yield of essential oil and significantly reduce the extraction time [7]. In addition, the combined method has been utilized to extract essential oil from various plants [4,8,9]. Although the ionic liquid assisted extraction of essential oil has been widely studied, little is known regarding ILs’ mechanism for enhancing the isolation efficiency of essential oil. For deeper understanding of the role of ILs, it is necessary to explore the mechanism of ILs in the essential oil process.

To understand the mechanism of ILs, some authors have speculated on the possible reasons for increase in extraction efficiency of essential oil [4,8,9]. Based on scanning electron microscopy (SEM) and infrared spectroscopy, it was deduced that ILs can destroy the cellulose structure in the cell wall. However, these were insufficient to comprehensively understand the mechanism of ILs. Theoretical calculations, such as atoms in molecules (AIM) [10], natural bond orbital (NBO) [11], and reduced density gradient (RDG) [12], are powerful tools to investigate the interaction between two molecules. Based on this method, the nature of interactions between ILs with chitobiose and *α*-cyclodextrin were explored with regard to dissolution mechanisms [13]. Moreover, theoretical calculations could be used to investigate the interaction of ILs with microwaves in extracting essential oil by microwave hydro-distillation [14]. It was demonstrated that theoretical calculation is an optional way to explore ILs’ mechanism in increasing extraction efficiency.

In this work, the extraction of essential oil from *Foeniculi fructus* was chosen as a benchmark for proposed methodologies due to its commercial value and biologic activities. *Foeniculi fructus*, listed in the Chinese Pharmacopoeia (ChP, 2020 edition) [15], is a commonly used medicinal plant. As principal active chemical compositions in *Foeniculi fructus*, the essential oil is widely used in the food and pharmaceutical industry [16].

Consequently, the purpose of this work is not only to optimize the extraction efficiency of essential oil from Fennel (*Foeniculi fructus*) by MILT-HD, but also to explore the mechanism wherein ILs improve extraction efficiency. Based on the DoE, multivariate analysis models were built. According to the multi-objective extracting process, the developed models were utilized to optimize the critical process parameters for enhancing extraction efficiency and yield. In order to explore the mechanism of ILs, density functional theory (DFT) was applied to reveal the mechanism using cellobiose as a model molecule. Based on non-covalent interaction analysis, the mechanism of ILs for enhancing extraction efficiency of essential oil was proposed. Moreover, the proposed mechanism was confirmed by scanning electron microscopy and Fourier transform infrared spectroscopy.

## 2. Experiment

### 2.1. Materials and Chemicals

*Foeniculi fructus* was brought from the local Chinese medicinal pharmacy in Beijing, China. Four ionic liquids containing 1-butyl-3-methylimidazolium bromide ([C4mim]Br, Lot number KCDLL06), 1-Octyl-3-methylimidazolium bromide ([C8mim]Br, Lot number ATW598), 1-butyl-3-methylimidazolium chloride ([C4mim]Cl, Lot number KCDGP56), 1-allyl-3-methylimidazolium chloride ([Amim]Cl, Lot number L1916039) were provided by Innochem Company (Beijing, China). Other reagents used for gas chromatography–mass spectrometry (GC–MS) analysis were purchased from Tianjin Kermel Chemical Reagent Co. Ltd., (Tianjin, China). The water used in the extraction process was deionized. 

### 2.2. Extraction Procedure of Essential Oil from Foeniculi Fructus

In this extraction procedure, a domestic Glanz microwave oven (Glanz Electrical Appliance Industrial Co., Ltd., Guangdong, China) with maximum radiation power of 800 W and irradiation frequency of 2.45 GHz was utilized to isolate the essential oil. A round hole was artificially modified on the top of the microwave oven to facilitate the connection of the flask and Clevenger apparatus. In this work, the microwave-assisted ionic liquids treatment followed by hydro-distillation (MILT-HD) was applied to extract the essential oil from *Foeniculi fructus*. The process consists of two stages, namely MILT and HD. In the MILT process, the *Foeniculi fructus* (10 g) and ILs were respectively weighted, and then added to a 250 mL reaction flask. Next, the reaction flask was symmetrically placed in a microwave oven. In order to collect the essential oil, the Clevenger apparatus was connected to the flask through a round hole at the top of the microwave. Based on the DoE, the sample mixtures were treated by ILs at different process parameters. In the HD stage, 60 mL deionized water was introduced into the reaction flask. The dark slurry produced from the MILT process was thoroughly mixed with deionized water, then heated on an electric stove (H22-X3, Hangzhou Joyoung Electrical Appliance Industrial Co., Ltd., Hangzhou, China). The electric stove was set to 100 °C for the hydro-distillation process.

For the control experiment, the *Foeniculi fructus* (10 g) and 60 mL deionized water were added to the 250 mL reaction flask. Then the electric stove set to 100 °C was used to heat the reaction flask connected with the Clevenger apparatus. The process of isolating the essential oil was performed until no more oil was collected. Anhydrous sodium sulfate was used to dehydrate the essential oil, and then the oil was stored in amber-colored vials at 4 °C until use. The yield of essential oil was calculated by Equation (1):

(1) $$Y_{\mathrm{eo}}\%=\frac{volume\;of\;essential\;oil(\mathrm{mL})}{weight\;of\;sample\;(g)}\times 100\%$$

### 2.3. DoE for the Extraction Process 

#### 2.3.1. Kinetic Model

In this study, in order to investigate the isolation process of essential oil, a first-order kinetic model [7] was applied to quantify the relationship between extraction time and extraction efficiency. The relevant equations were shown as follows:

(2) $$Y_{t}=Y_{\mathrm{eo}}[1-\exp(-k\times t)]$$

where *Y_t_* and *Y*_eo_ (%, mL·g^−1^) represent the yield of essential oil at time *t* (min) and at equilibrium, respectively. *k* (% min^−1^) indicates the mass transfer coefficient of the entire extraction process.

#### 2.3.2. DoE for MILT-HD

As a critical operation process in the whole extraction procedure, it is necessary to optimize the MILT process. In this operation unit, ILs ratio (*X*_1_), microwave irradiation time (*X*_2_), and microwave irradiation power (*X*_3_) were identified as critical process parameters (CPPs) based on a previously published study [2,9]. Consequently, it is vital to optimizing these three CPPs in this step. A Box-Behnken design was used to investigate the relationship between the CPPs and the response factors. According to preliminary experiments, the ILs ratio (*X*_1_), microwave irradiation time (*X*_2_), and microwave irradiation power (*X*_3_) were in the range of 50–90%, 2–4 min, and 10–30%, respectively. Table 1 listed 15 experiments based on the Box-Behnken design (BBD). The extraction yield and mass transfer coefficient of essential oil would be increased by assisting ILs. In previous researches, only yield of essential oil was a response factor, which could not systematically reflect the extraction efficiency. Hence, to comprehensively investigate the effects of CPPs on the extraction process, *Y*_eo_, the yield of essential oil at 90 min (*Y*_t90_), and *k* were response factors in this work. 

Based on the DoE, three MVA models were built through analysis of variance (ANOVA) evaluation. According to the developed quadratic reduced models, the main effects and interaction effects of operating conditions were investigated through the surface response plot. Moreover, a multi-objective optimization was used to obtain the optimum operating parameters for increasing the yield of essential oil and efficiency.

### 2.4. GC-MS Analysis of Essential Oil

In this paper, gas chromatography-mass spectrometry spectra were recorded using GCMS-QP2010 Ultra spectrometer (Shimadzu Corporation, Kyoto, Japan) with a gas chromatograph equipped with a WM-5MS capillary column (30 m × 0.25 mm × 0.25 μm film thickness), and a mass selective detector. 

Before the GC-MS analysis, the essential oil from *Foeniculi fructus* was diluted with ethyl acetate (1:100, *v*/*v*). The operating parameters of GC-MS were set as follows: The injector temperature was set as 250 °C. A 1 μL sample was injected into the GC column, and the splitting ratio was 1:20, helium gas flow rate 1 mL·min^−1^. The column temperature was programmed from 65 °C for 2 min, followed by a 5 °C·min^−1^ increased to 120 °C, then increased to 200 °C at a rate of 10 °C·min^−1^, finally increased to 250 °C at a rate 20 °C·min^−1^ for 1 min. The ion source temperature and scan range were set at 220 °C and 30–500 amu, respectively. The identification of essential oil compounds was implemented through a comparison of literature data, as well as by the comparison of their mass spectra fragmentation patterns with those of similar compounds stored in library NIST08.

### 2.5. Fourier Transforms Infrared Spectroscopy (FTIR)

After being extracted by various methods, the *Foeniculi fructus* was separated from the extraction solution and dried in the oven. The dried samples were milled and mixed with potassium bromide. In the process of IR measurement, the mixed powders were pressed into tablets for infrared scanning. Potassium bromide was used as the reference background. An IR spectrophotometer (Perkinelmer, PerkinElmer enterprise management (Shanghai) Co., Ltd., Shanghai, China) within 4000–400 cm^−1^ was used to scan the IR spectra of samples. The functional groups or chemical bonds of the various samples were qualitatively identified as per the IR spectra.

### 2.6. Scanning Electron Microscopy (SEM)

The *Foeniculi fructus* samples obtained in Section 2.5 and raw material samples were fixed on a metal disc and put on an ion sputtering apparatus (SC7620, Quorum, UK). Then, the surface morphological changes of the samples were observed using a scanning electron microscope (Quanta250, FEI, Hillsboro, OR, USA) with high vacuum conditions at a voltage of 15.0 KV.

### 2.7. Quantum Chemical Calculations

Cellulose, a macro-molecular polysaccharide, is one of the main components of the plant cell wall, containing several hundred to many thousands of *β* (1–4) linked *D*-glucose units [17]. As the basic cellulose unit, cellobiose was used to investigate the interaction between cellulose and ILs based on the density functional theory (DFT). In quantum computation, the M062X and 6–311 + G (d, p) theoretical levels were used to optimize the geometry of cellobiose and ILs, and calculate the interaction energy data. Since the whole system was neutral, the charge was set to 0. The multiplicity was set to 1 in the calculation process. After geometry optimization by calculation, no imaginary frequency was found in this paper. In order to study the interactions between cellobiose and ILs, the atoms in molecules (AIM), natural bond orbital (NBO), and reduced density gradient (RDG) were utilized to analyze the strength of non-covalent interactions based on the Multiwfn code [18] and Gaussian 09 software package [19]. In the NBO and RDG analysis, the isosurface was set to 0.05. The ranges of sign (λ2) *ρ* (au) were set to −0.05 to 0.05 in mapped RDG.

### 2.8. Data Analysis

The first-order kinetic curves were fitted by MATLAB 2009a platform (MathWorks, Natick, MA, USA). The Design-Expert 8.0 (State-Ease, Inc., Minneapolis, MN, USA) was used to develop the MVA models. All experimental data were expressed as means ± standard deviations.

## 3. Results and Discussion

### 3.1. Quantification of the Critical Operating Parameters Affecting the Extraction Process

**Figure 1:**
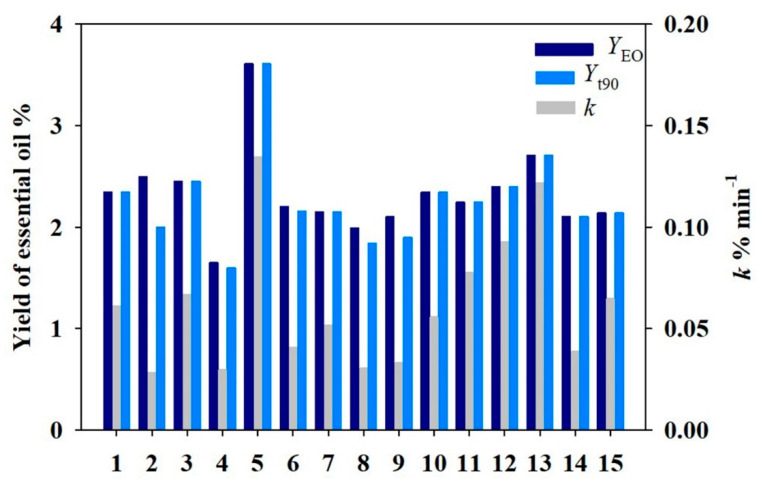
The extraction efficiency of essential oil in the DoE.

According to Supplementary Materials (Figure S1), [C4mim]Br was performed in MILT process based on the DoE listed in Table 1. The *Foeniculi fructus* essential oils were then extracted by the conventional HD process. Based on Equations (1) and (2), three critical response variables were computed. The result of the 15 experiments is displayed in Figure 1. It can be seen that the extraction efficiency of essential oils is different under various process parameters. Therefore, obtaining optimal process parameters is a valuable task.

#### 3.1.1. Critical Operating Parameters Affecting the *Y*_eo_

Based on the DoEs, the reduced quadratic model was developed. Furthermore, ANOVA was applied to analyze the MVA model. The best-fitted quadratic model was utilized to quantify the relationship between the operating parameters and *Y*_eo_ by comparing statistical indexes, like the correlation coefficient (R^2^) and probability value (*p*-value). Table 2 indicated that the linear term of *X*_2_, *X*_3_, interaction items of *X*_2_*X*_3_, and quadratic terms of *X*_1_^2^ were significant effects on the *Y*_eo_. The ANOVA results for the response surface quadratic method for *k* and *Y*_t90_ can be seen in Tables S1 and S2 in Supplementary Materials. The reduced quadratic model was expressed by code as follows:

(3) $$Y_{\mathrm{eo}}=2.46+0.25X_{2}+0.35X_{3}+0.39X_{2}X_{3}-0.24X_{1}{}^{2}$$

The determination coefficient (R^2^) and adj-R^2^ were equal to 0.8818 and 0.8346, respectively. The *p*-value of the model was 0.001. It indicated that the relationship between independent variables and *Y*_eo_ was highly significant. The *p*-value of “lack of fit” was 0.30, indicating that it was not significant. This implies that the MVA model was well fitted [20].

**Figure 2:**
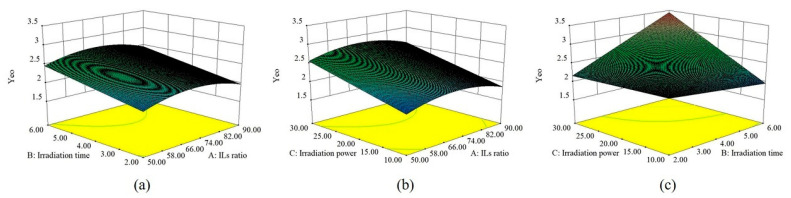
Response surface graphs of *Y*_eo_. (**a**) Interaction of ILs ratio and microwave irradiation time; (**b**) Interaction of ILs ratio and microwave power; (**c**) Interaction of irradiation time and microwave power.

Surface response plots, as a three-dimensional plot, were utilized to visualize the quadratic model. The surface response plots for *k* and *Y*_t90_ are shown in Figures S2 and S3 in Supplementary Materials. As exhibited in Figure 2, *Y*_eo_ increases and then decreases with increasing [C4mim]Br mass concentration at a given irradiation powder. It can be inferred that the increase in the mass concentration of ILs could significantly improve the dissolving capacity of the cellulose [2]. However, with the further increase of ILs concentration, the viscosity of IL increases, which reduces the mass transfer of the extraction process. Consequently, the extraction efficiency may decrease [8]. With the increase of power, *Y*_eo_ presented an upward trend. Because IL has excellent microwave absorbing capacity, it helps increase IL’s ability to penetrate plant tissue with increasing microwave power within a specific range [21], which results in destructing the cell wall and enhancing mass transfer [22]. As shown in Figure 2a, *Y*_eo_ increased with rise in the reprocess time. However, with further prolonging microwave time, *Y*_eo_ slightly reduces. There is no doubt that an appropriate treatment time is beneficial for dissolving the cell wall, while too a long reprocess time may lead to degradation of thermosensitive components.

#### 3.1.2. Multiple Objective Optimization and Verification

For a single response, it is easy to obtain the optimal operating process parameters using surface response design. In order to improve extraction efficiency, rather than only the yield of essential oil, this study optimizes three responses, including *Y*_eo_, *Y*_t90_, and *k*. Hence, a Derringer’s desirability function [23] was used to optimize three responses simultaneously. The overall desirability function *D* was calculated by Equation (4):

(4) $$D={\left\{d_{1}\times d_{2}\times d_{3}\times \cdots \times d_{m}\right\}}^{1/m}$$

where *d*_1_, *d*_2_, *d*_3_, ……, *d*_m_ represent the individual desirability function for each response. The optimal process variables of extracting essential oil for three responses were simultaneously investigated using the numerical optimization function of the Design-Expert software. Firstly, the three responses were set to a maximum value according to the goal. Then the optimal process conditions of extracting essential oil were given by the developed models. The optimum process conditions are listed in Table 3. Finally, in order to investigate the suitability of the reduced quadratic model for predicting the optimal process conditions, the verification experiments were implemented under the optimal process operating conditions. As can be seen from Table 3, the relative errors between the predicted and experimental results were within an acceptable range. It demonstrated that the reduced quadratic model could accurately predict the extraction efficiency of essential oil from *Foeniculi fructus*, indicating that the developed models were valid.

**Figure 3:**
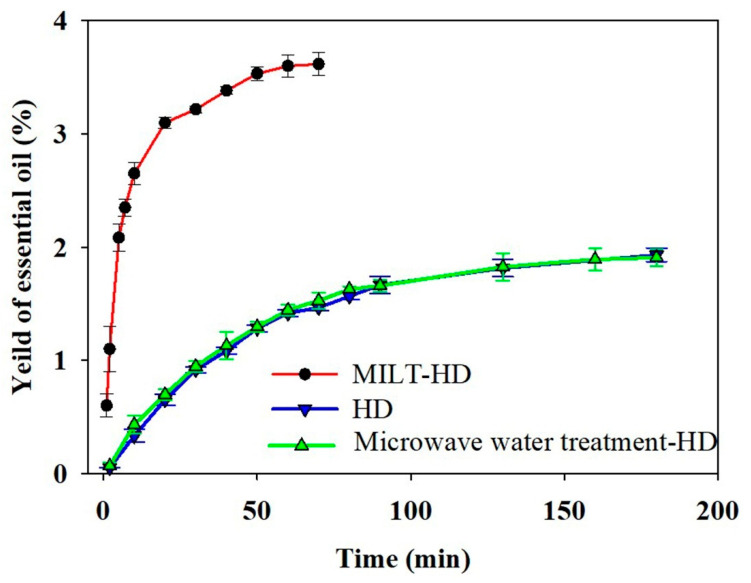
The extraction efficiency of essential oil from *Foeniculi fructus* using different methods.

To explore the effect of microwave irradiation, the microwave water treatment–HD (MWT–HD) process was the same as that of MILT–HD. Under the optimized conditions, water instead of ILs was used to extract the essential oil. It can be seen from Figure 3 that the extraction efficiency between MWT-HD and HD did not have a noticeable difference. This indicated microwave radiation of a short duration did not have a significant effect on the extraction process. Under the optimal conditions, the extraction efficiency of MILT–HD was significantly increased, indicating that MILT–HD is an efficient extraction technology for extracting essential oil [2].

### 3.2. GC-MS Analysis

**Figure 4:**
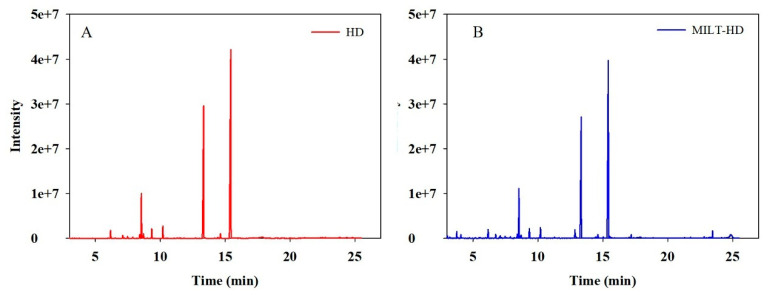
Total ion chromatogram of the essential oil obtained by different methods: (**A**) HD; (**B**) MILT–HD.

In the paper, GC-MS was applied to compare the component difference of essential oil between conventional HD and MILT-HD. Table 4 listed the essential oil components, retention index, molecular formula, molecular weight, and relative peak area. A chromatogram of the essential oil is shown in Figure 4. From Table 4, the identified chemical composition in HD mainly included *α*-pinene, *D*-Limonene, *γ*-terpinene, fenchone, estragole, anisic aldehyde, and anethole. This is consistent with previous studies [16,24,25]. The mass spectra of the main compounds and chemical structures were illustrated in Supplementary Materials (Figure S4). In order to achieve a more reliable qualitative analysis, the retention index and mass spectra of the main components were compared with data from the website [26] and literature [27], respectively. The components identified in the essential oil extracted by MILT-HD and HD accounted for 96.99% and 94.62%, respectively, of the total essential oil. It can be seen from Table 4 and Figure 4 that MILT–HD showed superiority in the number of essential oil components, as compared to that of the HD. Combined with the analysis in Figure 3, it demonstrated that the MILT–HD approach improved the yield of *Foeniculi fructus* essential oil and obtained more components.

### 3.3. Energy Demands and CO_2_ Emission

In this paper, the energy demands and environmental impact of MILT–HD and HD were evaluated by some indexes, such as time consumption, total electricity consumption, yield of essential oil per kilowatt–hour, and CO_2_ emission [28]. It can be seen from Table 5 that HD used 2.89 h to acquire 0.0193 mL/g of essential oil, while MILT–HD only required a shorter time to extract 0.0363 mL/g. The total energy consumed by HD (1.73 kW·h) was much greater than that of MILT–HD (0.741 kW·h). According to previous studies, one kW·h electricity consumption emits 800 g CO_2_ into the air [29]. From the perspective of environmental protection, MILT–HD is an environmentally friendly extraction technology, because it releases less CO_2_. Considering the yield of essential oil per kilowatt–hour, MILT–HD showed higher extraction efficiency compared with HD. The above results demonstrate that MILT–HD is an efficient extraction technique, with many advantages, which include low energy demands and low CO_2_ emission.

### 3.4. Structural Changes after Extraction

**Figure 5:**
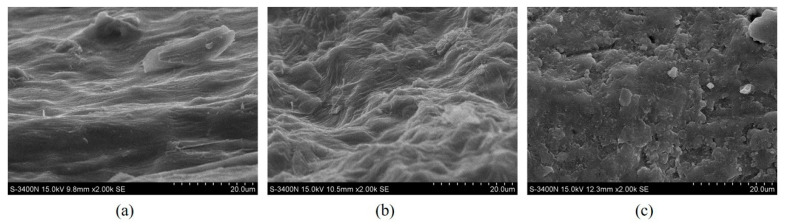
SEM images of the tested samples. (**a**) Sample of raw material; (**b**) Sample of HD; (**c**) Sample of MILT–HD.

According to the above analysis, MILT–HD can significantly improve the extraction efficiency of essential oils. In order to explore the reason for the same, the plant samples were examined by FTIR analysis and SEM to investigate structural changes. As can be seen from Figure 5, physical changes are noticeable on the *Foeniculi fructus*. The morphology of the MILT–HD sample was significantly different from that of the raw material. However, morphological integrity in the raw material and in the HD sample was almost retained, speculating that the cell walls were damaged [2], which led the target to take in extraction solution that could dissolve it [4].

**Figure 6:**
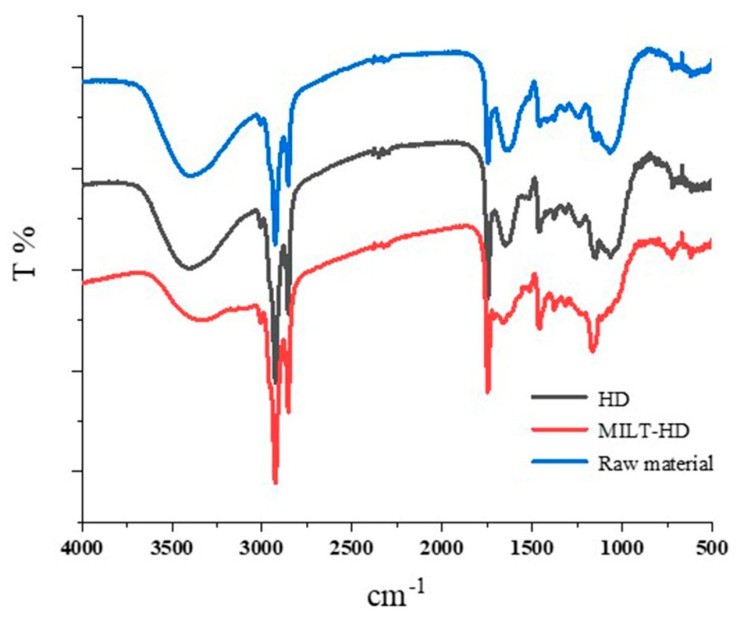
FTIR spectra of the tested samples.

The IR spectra was able to offer some important information for function groups or chemical bonds. It can be seen from Figure 6 that the signal intensity of the absorbance was not changed in the HD sample, as compared to those of the raw material of *Foeniculi fructus*. This demonstrates that the chemical structures of carbohydrate compounds were unbroken, indicating that the cell wall retained its integrity after the HD process. However, for the sample processed by MILT, the intensity of the absorbance at 3424 cm^−1^ (OH stretch) was decreased. This revealed that ILs could destroy the network of hydrogen bonds between the carbohydrate hydroxyl protons [2]. Therefore, it was easy for the essential oil in the cells to diffuse into the extraction solution. This may be an important reason why ILs improve extraction efficiency.

### 3.5. Interaction between Cellulose and ILs

According to IR spectroscopy and SEM, it was speculated that ILs could destroy the cell wall structure, but it was still unclear how ILs destroy the hydrogen bond structure of the cell wall. Because cellulose is the main component of plant cell walls, cellobiose, as the basic cellulose unit, was used as a model molecule to investigate the interaction with ILs.

#### 3.5.1. Geometries and Interaction Energy Analysis

**Figure 7:**
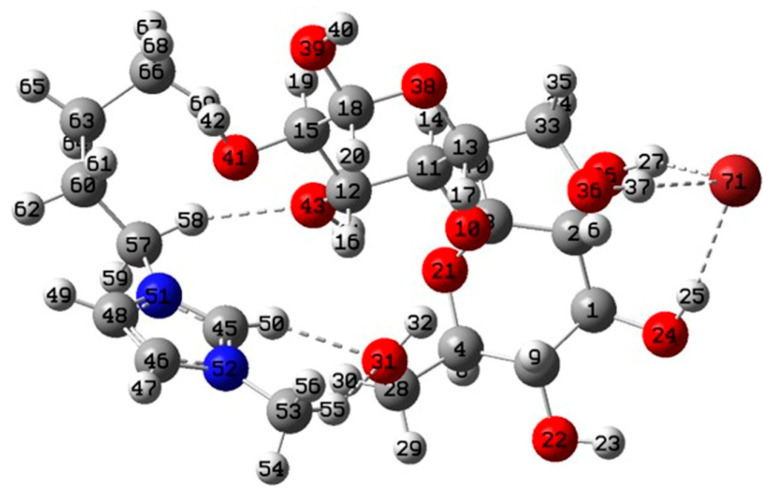
Geometry of the cellobiose/[C4mim]Br. Carbon: gray; oxygen: red; nitrogen: blue; bromine: dark brown.

The optimized structure of the cellobiose/BmimBr complex is shown in Figure 7. It can be seen that intermolecular hydrogen was formed between the cellobiose and the ILs—O(31)⋯H(58)-C(57) and Br(71)⋯O(26)-C(27). This indicated that both anion and cation could interact with cellobiose. The interaction energy was calculated as follows:

(5) $$\Delta E=E_{C/ILs}-(E_{C}+E_{ILs})$$

where E_C/ILs_, E_C_, and E_ILs_ represent the energies of cellobiose/ILs system, cellobiose, and ILs, respectively. According to the computation, the value of ΔE was equal to −43.83 KJ/mol, indicating that the cellobiose could be dissolved in the ILs [30]. This means that ILs are able to dissolve cellulose in the cell wall of *Foeniculi fructus*.

#### 3.5.2. AIM Analysis

To further investigate the mutual interaction between ILs and cellobiose, the AIM was used to reveal the non-covalent interaction. Table 6 provides the key topological parameters at the bonding critical point (BCP) of interaction between the ILs and the cellobiose. These parameters include electron density (*ρ*_BCP_), Laplacian (v^2^*ρ*_BCP_), curvature (λ), potential density (V_cp_), and the energy of hydrogen bond (E_HB_). The E_HB_ can be calculated by using the Espinosa-Molins-Lecomte equation [31], as shown below:

(6) $$E_{HB}=V_{cp}/2$$

and

(7) $$\nabla^{2}\rho_{BCP}=\lambda_{1}+\lambda_{2}+\lambda_{3}$$

where λ_1_, λ_2_, and λ_3_ stand for first, second, and third eigenvalues of the Hessian matrix.

It can be seen from Table 6 that the *ρ*_BCP_, v^2^*ρ*_BCP_ were within the ranges of 0.002–0.035 a.u and 0.024–0.139, respectively. This revealed that it was a closed shell interaction [30]. Generally, v^2^*ρ*_BCP_ is larger than 0, indicating the presence of a non-covalent interaction, such as hydrogen bond, van der Waals forces, and ionic bond. Moreover, one of three eigenvalues was positive and others were negative, demonstrating the chemical interaction between the two atoms. According to the classification of intermolecular hydrogen bonds, E_HB_ within −2.5 to −14.0 kcal/mol was weak to medium strength of H-bond [32]. It can be seen from Table 6 that the values of E_HB_ were larger than −14.0 kcal/mol. It revealed that the ILs and cellobiose system could form weak or weak to medium strength of H-bond.

#### 3.5.3. RDG Analysis

**Figure 8:**
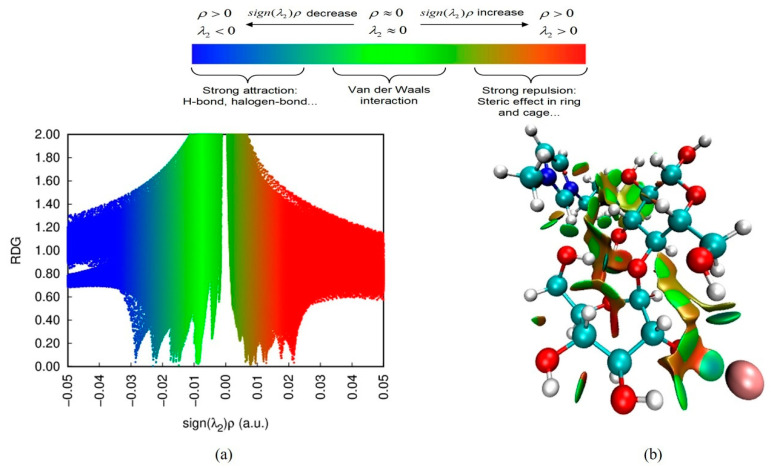
The RDG scatter plot (**a**) and sign (λ_2_) *ρ* mapped RDG isosurfaces (**b**) of cellobiose/[C4mim]Br.

The RDG was able to detect non-covalent interactions in real space based on the electron density and its derivatives [12]. In the plot of RDG, the low gradient spikes can reflect the interaction strength, and the λ_2_ can discern bonded (λ_2_ < 0) from nonbonded (λ_2_ > 0) interactions. According to the RGD plot, the pattern could be divided into three types: a large and negative value of sign (λ_2_) *ρ* means a strong attraction like H-bond, halogen bond; sign (λ_2_) *ρ* near-zero represents a weak interaction such as van der Waals interaction; and a large and positive sign (λ_2_) *ρ* indicates strong repulsion or steric effect [33]. In Figure 8, several gradient spikes appear in the negative region. It revealed that H-bonds between ILs and cellobiose were formed. The spikes near zero indicated that the cations from ILs and cellobiose might form van der Waals interaction [13]. The RDG isosurface could be used to reveal the weak interactions in real molecular space, as shown in Figure 8. It can be observed that the anions of ionic liquids mainly form hydrogen bonds (the light blue) with cellulose. In contrast, cations form van der Waals forces (the green region) with cellulose.

#### 3.5.4. NBO Analysis

NBO theory is implemented to analyze mutual interactions between the donor and acceptor of ILs and cellobiose. It is beneficial for investigating intermolecular bonding or interaction among bonds. On the NBO basis, the second-order perturbation energies (E2) could be used to evaluate the strength of the interactions. The E(2) can be calculated by the below equation [34,35]:

(8) $$E(2)=q_{i}\frac{F^{2}{}_{\left(ij\right)}}{\varepsilon_{j}-\varepsilon_{i}}$$

where *q_i_* represents the occupancy of donor orbital. *F*_(*ij*)_ is the off-diagonal NBO matrix element. The *ε_i_* and *ε_j_* are diagonal elements.

**Figure 9:**
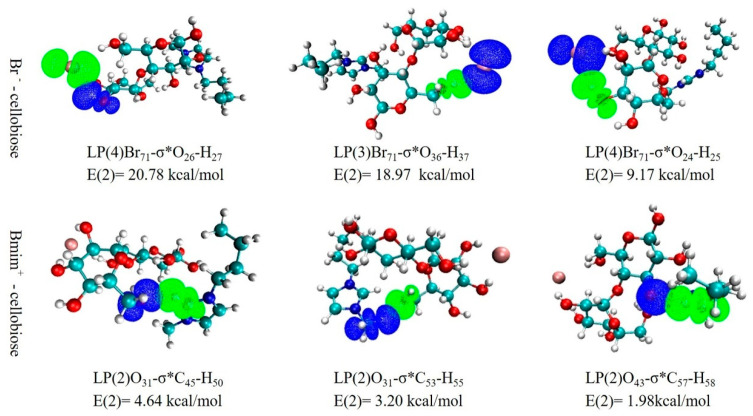
3D overlap images for donor-acceptor orbital interactions.

Figure 9 displays a 3D image of the orbital interaction between the ILs and the cellobiose. It can be found that the E(2) of the intermolecular orbital formed by anion of ILs and cellobiose is higher than that of the cation of ILs and cellobiose. The large E(2) indicates that there is a strong interaction between the electron donor and the electron acceptor, and the larger extent of conjugation of the entire system [36]. For example, the E(2) value of LP(4)Br_71_-σ*O_26_-H_27_ is 20.78 kcal/mol. This illustrates that the Br^−^ could effectively interact with the cellobiose. Consequently, it can be speculated that the anion of ILs plays a vital role in dissolving the cellulose.

**Figure 10:**
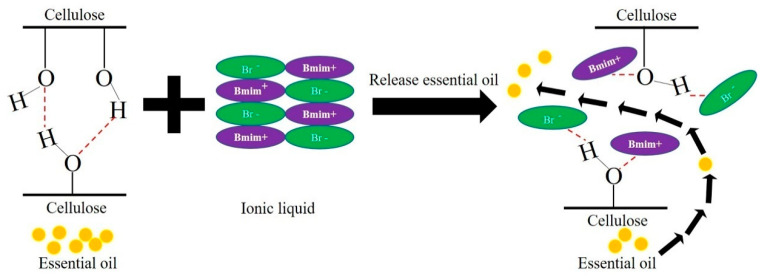
Graphic mechanism of ILs to enhance extraction efficiency.

In summary, based on the above analysis, the mechanism wherein ILs enhance the extraction efficiency of essential oil is that ILs can form an intense non-covalent bond interaction with the cellulose. The formation of an H-bond and van der Waals force between ILs and cellulose could impair the strength of H-bond in the cellulose. It helped destroy the network hydrogen bond structure of the cellulose. Consequently, it improves the permeability of the cell wall by destroying its integrity, leading to rapid leaking of essential oil from cells without slow permeation [37], as shown in Figure 10. It is conducive to enhance the release rate of essential oil and to promote the complete escape of the essential oil from the cells [4].

## 4. Conclusions

In this study, DoEs and MVA were used to evaluate the process of extracting essential oil using MILT-HD comprehensively. A multi-objective optimization was established to obtain optimum operating parameters for extracting essential oil from *Foeniculi fructus*. The optimal process variables were able to accelerate the isolation of essential oil from *Foeniculi fructus* and enhance the yield. Consequently, MILT–HD was found to be an efficient extraction technology. The quantum chemical calculation revealed the mechanism wherein ILs improve extraction efficiency. Based on infrared spectroscopy and SEM characterization, the proposed mechanism was confirmed.

However, there are some limitations in this study. For example, it is necessary to study whether essential oil properties have changed, such as pharmacological activity and antioxidant activity.

## Figures and Tables

**Table 1:** Box–Behnken experimental design matrix for the extraction of essential oil during the MILT process.

Runs	Process Parameters		
	*X*_1_ (%)	*X*_2_ (min)	*X*_3_ (%)
1	70	4	20
2	50	6	20
3	70	4	20
4	50	4	10
5	70	6	30
6	70	2	10
7	90	2	20
8	50	2	20
9	70	6	10
10	70	4	20
11	90	6	20
12	50	4	30
13	90	4	30
14	90	4	10
15	70	2	30

**Table 2:** ANOVA results for the response surface quadratic method for *Y*_eo_.

Source	Sum of Square	DF	Mean Square	*F* Value	*p*-Value	Significance
Model	2.29	4	0.57	18.66	0.0001	Significant
*X_2_*	0.48	1	0.48	15.74	0.0027	Significant
*X*_3_	0.98	1	0.98	31.85	0.0002	Significant
*X_2_ X*_3_	0.62	1	0.62	20.08	0.0012	Significant
*X*_1_^2^	0.21	1	0.21	6.96	0.0248	Significant
Residual	0.31	10	0.031			Significant
Lack of fit	0.30	8	0.037	10.49	0.0899	Not significant
R^2^	0.8818					

**Table 3:** Predicted and experimental values of the responses obtained under optimal extraction conditions.

	*X*_1_ (%)	*X*_2_ (min)	*X*_3_ (%)	*Y*_eo_ (%)	*Y*_t90_ (%)	*k* % min^−1^	Desirability
Predicted	73.19	6.00	30.00	3.437	3.440	0.1358	0.943
Experimental	73.20	6.00	30.00	3.633 ± 0.112	3.633 ± 0.112	0.1447 ± 0.0088	
RE (%)				5.70	5.61	6.55	

Note: RE (%) represents relative error.

**Table 4:** Key data for the major components of the *Foeniculi fructus* essential oil.

No.	Components	Retention Index	Molecular Formula	Molecular Weight	Relative Peak Area (%)	
					MD	MILT-HD
1	Acetic acid, butyl ester	785	C6H12O2	116	ND	0.68
2	Furfural	831	C5H4O2	96	ND	0.49
3	*α*-pinene	948	C10H16	136	0.95	1.25
4	*D*-Limonene	1018	C10H16	136	6.85	8.32
5	*γ*-terpinene	998	C10H16	136	1.36	1.53
6	Fenchone	1121	C10H16O	152	1.84	1.82
7	1-Butylimidazole	1013	C7H12N2	124	ND	2.07
8	Estragole	1172	C10H12O	148	31.28	28.71
9	Anisic aldehyde	1171	C10H12O	148	0.76	0.83
10	Anethole	1190	C10H12O	148	53.95	48.29
11	Palmitic acid, methyl ester	1878	C17H34O2	270	ND	0.63
Total identified peak area (%)					96.99	94.62

**Table 5:** Comparison of the economic value and environmental impact of different extraction approaches.

	MILT–HD		HD
	Pretreatment	Hydrodistillation	Hydrodistillation
Heating method	Microwave	Electric stove	Electric stove
Effective electric power (W)	390	600	600
Time consumption (h)	0.100	1.17	2.89
Electricity consumption (kW·h)	0.0390	0.702	1.73
Total electricity consumption (kW·h)	0.741		1.73
Yield of essential oil (mL/g)	0.0363		0.0193
Yield of essential oil per kilowatt hour (mL/g/(kW·h))	0.0490		0.0112
Environmental impact (g CO_2_ emission)	592.8		1384

**Table 6:** Topological parameters at BCPs for cellobiose/ILs.

H-Bond	*ρ*_BCP_ (a.u)	v^2^*ρ*_BCP_ (a.u)	V_cp_ (a.u)	λ_1_	λ_2_	λ_3_	E_HB_ (Kcal/mol)
Br_71_⋯C_24_-H_25_	0.0166	0.0448	−0.0085	−0.0150	0.0750	−0.0152	−2.6590
Br_71_⋯C_36_-H_37_	0.0230	0.0568	−0.0137	0.1044	−0.0233	−0.0243	−4.3095
Br_71_⋯C_26_-H_27_	0.0221	0.0569	−0.0135	−0.0218	−0.0212	0.0999	−4.2372
O_31_⋯C_53_-H_55_	0.0145	0.0518	−0.0094	−0.0143	−0.0162	0.0822	−2.9492
O_31_⋯C_45_-H_50_	0.0181	0.0664	−0.0122	0.1065	−0.0211	−0.0191	−3.8133
O_43_⋯C_45_-H_50_	0.0132	0.0460	−0.0087	−0.0108	−0.0123	0.0691	−2.7446
O_41_⋯C_60_-H_61_	0.0094	0.0308	−0.0061	0.0454	−0.0068	−0.0078	−1.9123
O_21_⋯C_43_-H_44_	0.0157	0.0561	−0.0107	0.0894	−0.0173	−0.0161	−3.3708

## Data Availability

The data presented in this study are available on request from the corresponding author. The data are not publicly available due to privacy.

## Supplementary Materials

The following are available online. Figure S1. Effects of different ILs on the yield of essential oil. Table S1. ANOVA results for response surface quadratic method for *k*. Table S2. ANOVA results for response surface quadratic method for *Y*_t90_. Figure S2. Response surface graphs of *k*. (a) Interaction of ILs ratio and microwave irradiation time; (b) Interaction of ILs ratio and microwave power; (c) Interaction of irradiation time and microwave power. Figure S3. Response surface graphs of *Y*_t90_. (a) Interaction of ILs ratio and microwave irradiation time; (b) Interaction of ILs ratio and microwave power; (c) Interaction of irradiation time and microwave power. Figure S4. The mass spectra and chemical structures of the main compounds ((a) *α*-pinene; (b) *D*-Limonene; (c) *γ*-terpinene; (d) Fenchone; (e) Estragole; (f) Anisic aldehyde; (g) Anethole).

## Sample Availability

Samples of the compounds are not available from the authors.
