# Supplementary material for: Study on the Mechanism of Ionic Liquids Improving the Extraction Efficiency of Essential Oil Based on Experimental Optimization and Density Functional Theory: The Fennel (Foeniculi fructus) Essential Oil Case
Source: Molecules. 2021 May 26;26(11):3169. doi: 10.3390/molecules26113169 (PMC8197825; doi:10.3390/molecules26113169)
Supplement: Supplementary file 1 [file molecules-26-03169-s001.zip › molecules-1216478-supplementary.pdf]

Supplementary materials

1. Choosing optimal ILs for MILT

Imidazoline salts were widely used in the extraction process. In this paper, the Y_{eo} was used as an index to evaluate the extraction performance of 1-alkyl-3-methylimidazolium-type ILs with various anions and alkyl chain lengths. For the ionic liquid screening process, extraction parameters were set up as follows: irradiation power 20%, irradiation time 4 min, mass concentration of ILs 70% for the MILT process. The results could be seen from Figure S1. In the above operating conditions, compared with conventional HD process, the MILT-HD could obviously enhance essential oil from *Foeniculi fructus*. However, there was no evident difference among various ILs. In this work, the price of ILs is considered as an important aspect, which may have a significant impact on the final large-scale application. Hence, [C4mim]Br with low cost was choice as the optimal IL in this paper for the subsequent separation of essential oil from *Foeniculi fructus*.

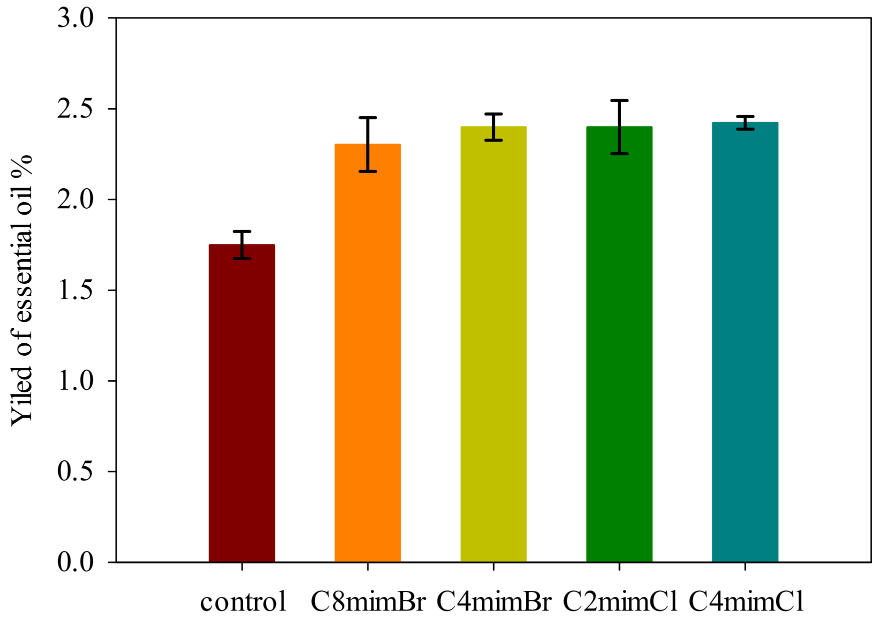

Figure S1. Effects of different ILs on the yield of essential oil

Table S1. ANOVA results for response surface quadratic method for *k*

Source	Sum of square	DF	Mean square	F value	p-value	Significance
Model	0.014	5	2.83E-003	23.50	< 0.0001	Significant
X_1	1.47E-003	1	1.47E-003	12.21	0.0068	Significant
X_2	9.25E-004	1	9.25E-004	7.67	0.0218	Significant
X_3	9.24E-003	1	9.24E-003	76.71	< 0.0001	Significant
X_2 X_3	1.51E-003	1	1.51E-003	12.51	0.0064	Significant
X_1^2	1.01E-003	1	1.01E-003	8.42	0.0176	Significant
Residual	1.09E-003	9	1.21E-004			Significant
Lack of fit	1.02E-003	7	1.46E-004	4.80	0.1832	Not significant
R^2	0.9289					

Table S2. ANOVA results for response surface quadratic method for Y_{t90}

Source	Sum of square	DF	Mean square	F value	p-value	Significance
Model	2.76	5	0.55	24.55	< 0.0001	Significant
X_1	0.24	1	0.24	10.50	0.0102	Significant
X_2	0.27	1	0.27	11.96	0.0072	Significant
X_3	1.20	1	1.20	53.33	< 0.0001	Significant
X_2 X_3	0.74	1	0.74	32.91	0.0003	Significant
X_1^2	0.32	1	0.32	14.04	0.0046	Significant
Residual	0.20	9	0.022			Significant
Lack of fit	0.20	7	0.028	7.80	0.1183	Not significant
R^2	0.9317					

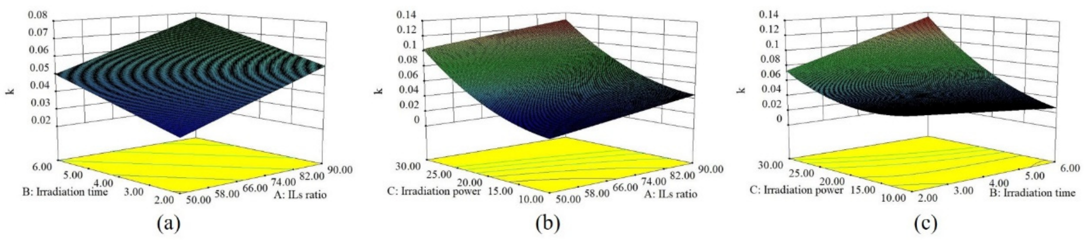

Figure S2. Response surface graphs of k. (a) Interaction of ILs ratio and microwave irradiation time; (b) Interaction of ILs ratio and microwave power; (c) Interaction of irradiation time and microwave power

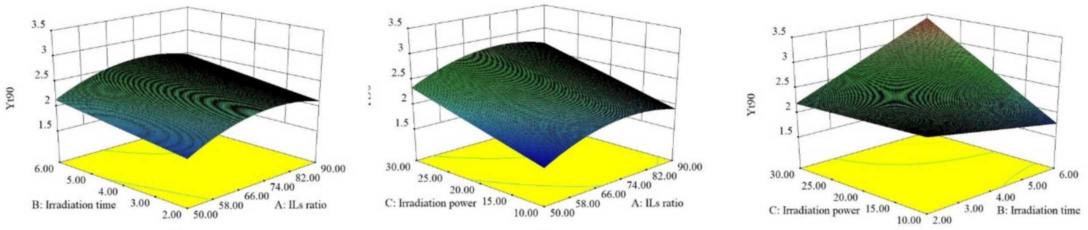

(a) (b) (c)

Figure S3. Response surface graphs of Y_{t90}. (a) Interaction of ILs ratio and microwave irradiation time; (b) Interaction of ILs ratio and microwave power; (c) Interaction of irradiation time and microwave power

Figure S4. The mass spectra and chemical structures of the main compounds ((a) *α*-pinene; (b) *D*-Limonene; (c) *γ*-terpinene; (d) Fenchone; (e) Estragole; (f) Anisic aldehyde; (g) Anethole).

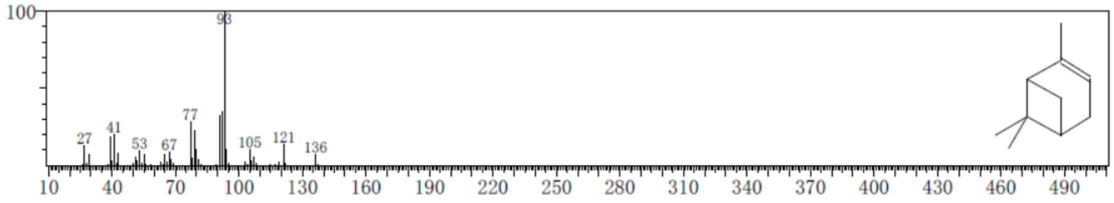

(a) *α*-pinene

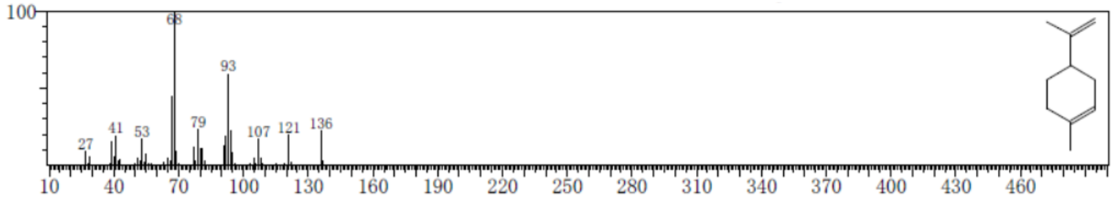

(b) *D*-Limonene

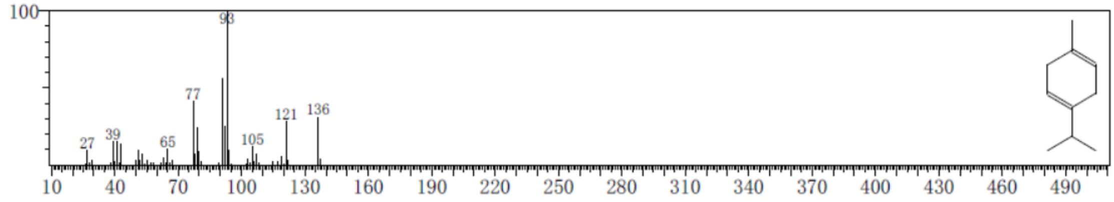

(c) *γ*-terpinene

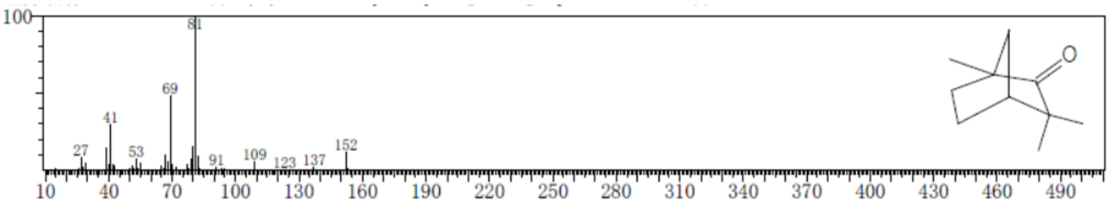

(d) Fenchone

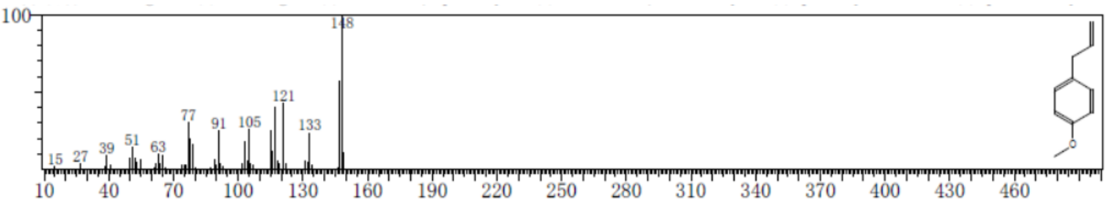

(e) Estragole

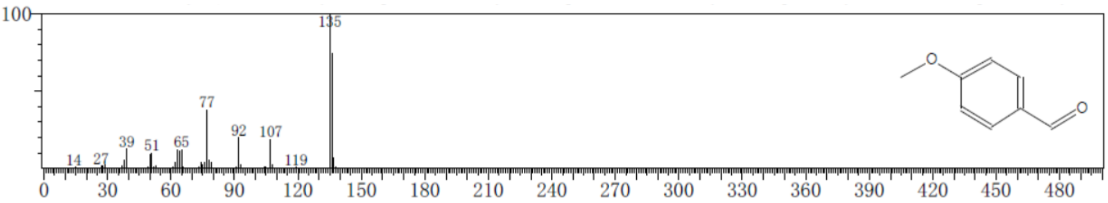

(f) Anisic aldehyde

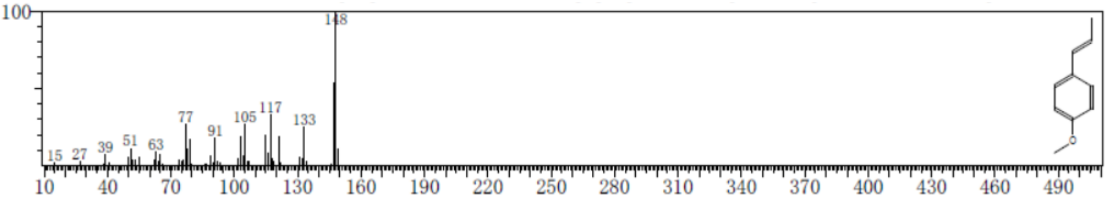

(g) Anethole
